# Redefinition of the toll-like receptor repertoire in *Ciona robusta* through genomic, structural, and expression analyses

**DOI:** 10.3389/fcimb.2025.1716256

**Published:** 2026-01-06

**Authors:** Akira Shiraishi, Shin Matsubara, Sakura Kikuchi, Kanako Hisata, Noriyuki Satoh, Larry J. Dishaw, Honoo Satake

**Affiliations:** 1 Bioorganic Research Institute, Suntory Foundation for Life Sciences, Kyoto, Japan; 2 Marine Genomics Unit, Okinawa Institute of Science and Technology Graduate University, Onna, Japan; 3 Currently Centrosome Dynamics and Evolution Group, Okinawa Institute of Science and Technology Graduate University, Onna, Japan; 4 Morsani College of Medicine - Pediatrics, Children’s Research Institute, University of South Florida, St. Petersburg, FL, United States

**Keywords:** ascidian, *Ciona robusta*, domain, structure, toll-like receptor, leucine-rich repeat

## Abstract

**Background:**

Toll-like receptors (TLRs) are essential components of innate immunity, mediating the recognition of pathogen-associated molecular patterns (PAMPs) through extracellular leucine-rich repeat (LRR) domains and initiating signaling via intracellular Toll/interleukin-1 receptor (TIR) domains. In the ascidian *Ciona robusta*, two canonical TLRs (CiTLR1 and CiTLR2) and several putative TLR-like genes (TLR3, -4, -6, -7, -13) have been annotated; however, their authenticity has remained uncertain due to limited structural and functional validation.

**Methods:**

we systematically reanalyzed the *Ciona* genome using the latest nearly complete assembly (HT genome) in combination with domain prediction, three-dimensional structural modeling, and transcriptomic expression profiling.

**Results:**

Genomic mapping and sequence comparison demonstrated that TLR13 is identical to CiTLR1, while TLR3, -6, and -7 lack a complete TIR domain, indicating that these are not canonical TLRs. We further identified a novel TLR gene, CiTLRs1, located approximately 42 kb from CiTLR1 on chromosome 14, which encodes all essential structural features including LRR and TIR domains. AlphaFold3 structural predictions confirmed that CiTLR1, CiTLR2, and CiTLRs1 possess canonical solenoid LRR folds and typical TIR domain architectures. In addition, we found no convincing evidence that CiTLR3, CiTLR6, or CiTLR7 function as soluble TLRs. Transcriptomic analyses revealed distinct tissue-specific expression profiles of these genes, suggesting nonredundant immune functions.

**Conclusions:**

Our findings revise the repertoire of bona fide TLRs in *Ciona* to three (CiTLR1, CiTLR2, CiTLRs1) and emphasize the risk of overestimating TLR diversity based solely on sequence homology without domain and functional validation. This work refines the structural and functional landscape of ascidian TLRs.

## 1 Introduction

Innate immunity serves as the primary defense mechanism against microbial invasion. In mammals, Toll-like receptors (TLRs) play central roles in initiating immune responses and serving essential regulatory functions that help mediate adaptive immune responses. TLRs are type I transmembrane proteins characterized by extracellular leucine-rich repeat (LRR) motifs, and an intracellular Toll/interleukin-1 receptor (TIR) domain, which are responsible for the specific recognition of pathogen-associated molecular patterns (PAMPs) and subsequent activation of downstream signaling cascades via adaptor proteins such as myeloid differentiation primary response (MyD)88 and TIR-domain-containing adapter-inducing interferon (TRIF), respectively (Takeda and Akira, 2015). The respective TLRs recognize distinct microbial and/or viral components and nucleic acids, and their activation leads to the production of inflammatory cytokines, chemokines, and interferons (Takeda and Akira, 2015).

Over the past 15 years, the TLR and its related genes have been detected in a wide variety of invertebrates, lacking traditionally recognized acquired or adaptive immune components and mechanisms. The absence of an adaptive immune system in invertebrates underscores the central importance of innate immunity systems in host defense (Nonaka and Satake, 2010; Longo et al., 2021; Perveen et al., 2025). However, most investigations of invertebrate TLRs have been limited to sequence homology searches based on genomic or transcriptomic data, or to immunohistochemical analyses employing antibodies whose specificity has not been validated—for example, using antibodies raised against human TLRs to detect putative invertebrate TLRs (Lauriano et al., 2021; Alesci et al., 2022; Bisanti et al., 2024).

In ascidians, which are invertebrate chordates and the sister group of vertebrates, various components of innate immunity have been identified, including mannose-binding protein-like collectins, galectins, phenoloxidase, complement pathways, chitin-binding proteins, antimicrobial peptides, and hemocytic peptides (Nonaka and Satake, 2010; Dishaw et al., 2011; Satake and Sekiguchi, 2012; Dishaw et al., 2016; Ohtsuka and Inagaki, 2020; Longo et al., 2021; Matsubara et al., 2024; Perveen et al., 2025; Natarajan et al., 2025). To date, TLRs or their candidate genes, such as CiTLR1, -2, -3, -4, -6, -7, and -13, have been annotated in *Ciona robusta* (previously classified as *Ciona intestinalis* and currently also referred to *Ciona intestinalis* Type A) (Sasaki et al., 2009; Satake and Sekiguchi, 2012; Lauriano et al., 2021; Alesci et al., 2022; Bisanti et al., 2024). In a previous study, we elucidated the full-length sequences, PAMP specificities, and subcellular localizations of CiTLR1 and CiTLR2, and demonstrated that both of the CiTLRs activated the canonical MyD88–NF(nuclear factor)-κB signaling cascades in response to PAMPs (Sasaki et al., 2009; Satake and Sasaki, 2010; Nonaka and Satake, 2010; Satake and Sekiguchi, 2012). Together with the identification of homologous genes encoding factors relevant to the TLR signaling pathway, such as the key associated adaptor MyD88 and the major transcriptional factor NF-κB (**Supplementary Figure 1**, in the *Ciona* genome (Azumi et al., 2003; Satake and Sekiguchi, 2012), these findings provided evidence that TLRs are phylogenetically and functionally conserved in *Ciona* and that CiTLR1 and -2 exhibit hybrid features in terms of PAMP recognition and intracellular localization, compared with mammalian TLRs (Sasaki et al., 2009; Satake and Sasaki, 2010; Satake and Sekiguchi, 2012).

In contrast, the remaining *Ciona* TLR candidates (CiTLR3, -6, -7, and -13) have not been thoroughly investigated for their structural organization nor functions. In the present study, we reanalyzed the previously reported *Ciona* TLR candidates, and demonstrate that only CiTLR1 and CiTLR2 possess the complete domain architecture consistent with bona fide TLRs. Additionally, we identified a novel TLR-like gene, CiTLRs1, which contains all essential structural features, including LRR, a transmembrane domain, and a TIR domain.

## 2 Materials and methods

### 2.1 Putative TLRs search on the *Ciona* genome

The amino acid sequences of *Ciona* TLRs and the human proteins involved in the downstream cascades were collected from Genbank and Uniprot, respectively. The corresponding genes in nearly complete genome of *Ciona robusta* (Satou et al., 2019) were detected using Blastp searches (version 2.13.0, National Center for Biotechnology Information Bethesda, MD, USA) with an E-value cut-off of 10^-10^. The sequence motifs (a signal peptide, LRRs, and/or a TIR domain) were detected using TMHMM, SignalP, SUPERFAMILY, PANTHER, and SMART as implemented in InterProScan version 6 (Blum et al., 2025) (https://www.ebi.ac.uk/interpro/download/InterProScan/).

### 2.2 Alignments of nucleotide or amino acid sequences

The nucleotide or amino acid sequences were aligned using CLUSTAL 2.1 (http://www.clustal.org/download/2.1/). IUB and BLOSUM matrix were used for calculating alignment scores for nucleotide sequence alignment, respectively.

### 2.3 Prediction of putative TLRs’ tertiary structures

The amino acid sequences for six putative TLRs were subjected to structure model prediction using AlphaFold 3 server (Abramson et al., 2024) (https://alphafoldserver.com/) with default parameters and the models with the lowest energies were used as predicted structures. Visualization and alignment of the protein models was performed using UCSF Chimera X (Meng et al., 2023)) (https://www.cgl.ucsf.edu/chimerax/).

### 2.4 Gene expression profile analysis

RNA-seq reads of *Ciona* tissues (PRJNA731286) were obtained from the Sequence Read Archive (SRA) and mapped to the nearly complete genome assembly of *Ciona robusta* (Satou et al., 2019) using HISAT2 version 2.2.0 (https://github.com/DaehwanKimLab/hisat2). Transcript abundances, expressed as TPM, were calculated using StringTie version 2.2.1 (http://ccb.jhu.edu/software/stringtie).

### 2.5 Collection and preparation of hemocytes from *C. robusta*

Adult ascidians (*C. robusta*) were obtained from the Maizuru Fisheries Research Station of Kyoto University through the National BioResource Project (NBRP), and were maintained in natural seawater with continuous aeration at room temperature for two days prior to hemocyte collection. Hemolymph was collected from the pericardium using a 20-gauge needle attached to a 1-mL syringe. Two samples were obtained from two individual animals and used to generate separate libraries. An aliquot of 100 µL of hemolymph was diluted with 900 µL of hemocyte buffer (11 mM KCl, 43 mM Tris-HCl, pH 7.5, 0.475 M NaCl; osmolarity ~975 mOsm kg^-^¹) and gently mixed by pipetting. The cell suspension was centrifuged at 300 × g for 5 min at 15 °C. After washing twice with hemocyte buffer, the hemocytes were resuspended in an equal volume of fresh buffer. Trypan blue staining confirmed >90% cell viability in all samples.

### 2.6 Library preparation, single-cell RNA sequencing (scRNA-seq), and data analysis

Single-cell suspensions were processed following the manufacturer’s protocol for the Chromium Next GEM Single Cell 3′ Reagent Kit v3.1 (Dual Index) (10x Genomics, Pleasanton, CA, USA). Target cell recovery was set to 5,000 cells per sample. Encapsulation and barcoding were performed using the Chromium Controller (10x Genomics). Libraries were constructed according to the manufacturer’s instructions. scRNA-seq was performed at the Sequencing Section of Core Facilities at Okinawa Institute of Science and Technology Graduate University (OIST) using the Illumina NovaSeq 6000 platform (Illumina, San Diego, CA, USA). The resulting fastq files were processed with Cell Ranger 9.0.1 (10x Genomics) using the *Ciona robusta* genome reference package HT.RefwMG0 (https://ghost.zool.kyoto-u.ac.jp/default_ht.html) (Satou et al., 2022). Downstream analyses were performed in R 4.5.1 using the following packages: *Seurat* (http://satijalab.org/seurat/), *ggplot2*(https://ggplot2.tidyverse.org/), and *patchwork* (https://patchwork.data-imaginist.com/). The integrated data were normalized, variable features were identified, and scaling and principal component analysis (PCA) were performed. After PCA, datasets were integrated using canonical correlation analysis, and clustering and UMAP visualization were performed based on the integrated dimensions. Gene expression patterns of CiTLRs, marker genes, and immune-related genes were subsequently visualized using dot plots.

## 3 Results

### 3.1 Genomic re-analyses of *CiTLRs* on the latest *Ciona* genome

**Figure 1:**
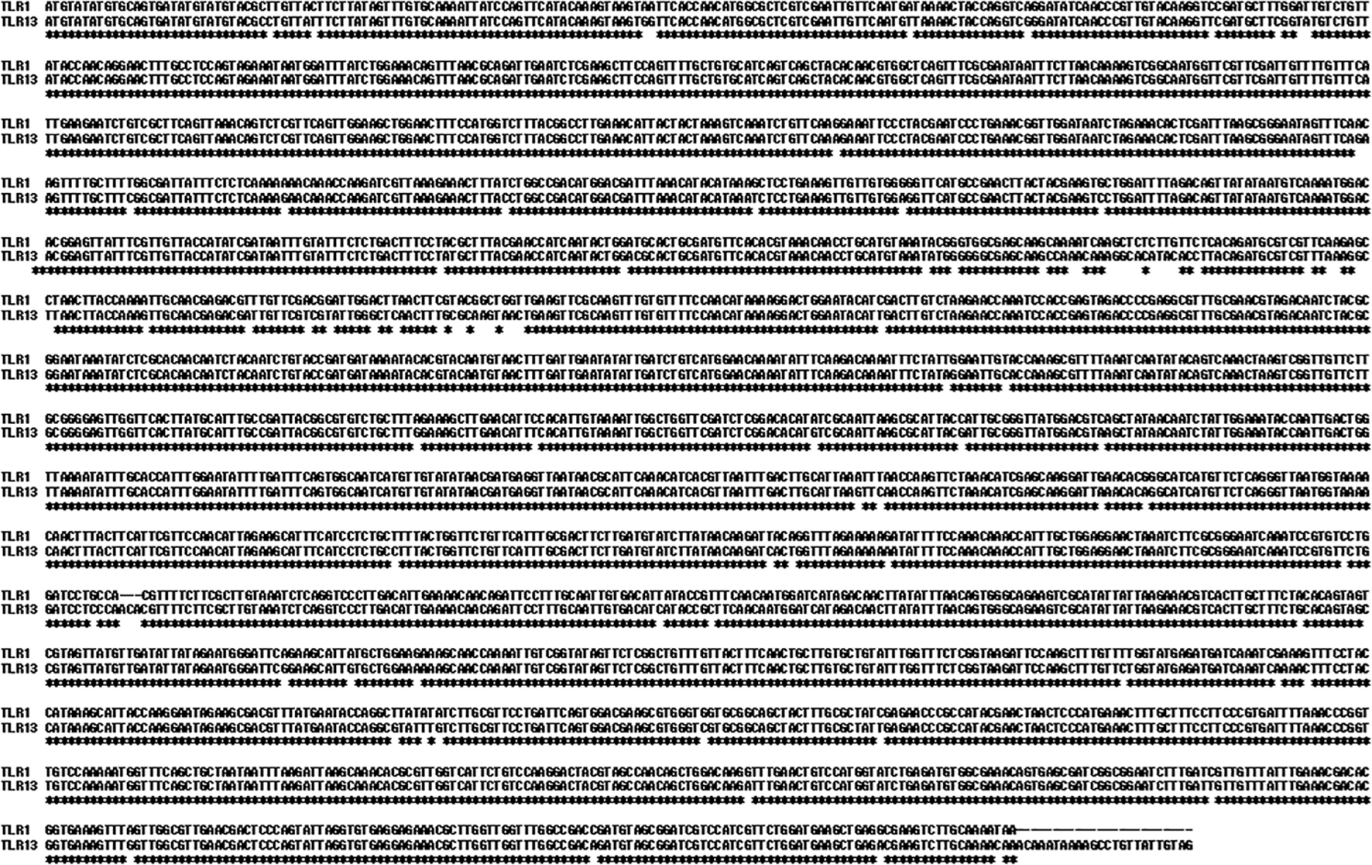
Sequence alignments of *Ciona* TLR1 and TLR13 genes. Identical nucleotides are marked by *.

Initially, we used BLASTp to search the genomic loci and homologous proteins of the *Ciona* genes with putative annotation as CiTLR (*TLR1, 2, 3, 6, 7, 13*) against the latest *Ciona* genome assembly, the HT genome (http://ghost.zool.kyoto-u.ac.jp/default_ht.html), which represents a nearly complete assembly. **Table 1** summarizes the gene identity, the loci, and sequence similarity of the respective CiTLRs. *CiTLR2* and the putative *CiTLR3, -6*, and *-7* were each mapped to distinct genes in the HT genome—KY21.Chr8.726, KY21.Chr13.295, KY21.Chr4.574, and KY21.Chr1.44, respectively—with complete sequence coverage and high sequence identity (98.68–99.57%). Sequence comparisons at both the nucleotide and predicted amino acid levels revealed >94% identity between CiTLR1 and CiTLR13 (**Figure 1**). In addition, both were mapped to the same genomic region, KY21.Chr14.7 (**Table 1**), indicating that they represent alternative annotations of the same gene locus. Taken together, these results indicate that CiTLR13 is a redundant annotation of CiTLR1 and that there is no distinct gene corresponding to CiTLR13. We therefore hereafter refer to CiTLR13 as CiTLR1.

**Table 1:** Blast search for TLR-like genes in the HT genome.

Query in previous annotation in NCBI	Query coverage	Hit gene in HT genome	Redefined name	Percent identity	P-value
TLR1 (NP_001159599.2)	100.0%	KY21.Chr14.7	CiTLR1 (and -13)	96.46%	0.0
TLR1 (NP_001159599.2)	47.42%	KY21.Chr14.65	CiTLRs1	98.96%	0.0
TLR2 (NP_001159600.1)	100.0%	KY21.Chr8.726	CiTLR2	98.86%	0.0
TLR13 (XP_002120520.2)	100.0%	KY21.Chr14.7	CiTLR1 (and -13)	99.51%	0.0
TLR13 (XP_002120520.2)	47.42%	KY21.Chr14.65	CiTLRs1	97.59%	0.0
TLR3 (XP_002120237.1)	100.0%	KY21.Chr13.295	*CiTLR3	98.68%	0.0
TLR6 (XP_002130253.1)	100.0%	KY21.Chr4.574	*CiTLR6	99.57%	0.0
TLR7 (NP_001231972.1	100.0%	KY21.Chr1.44	*CiTLR7	98.79%	0.0

Variant information for the hit protein is shown in brackets. * Indicates CiTLR-like genes that lack a complete TIR domain.

**Figure 2:**
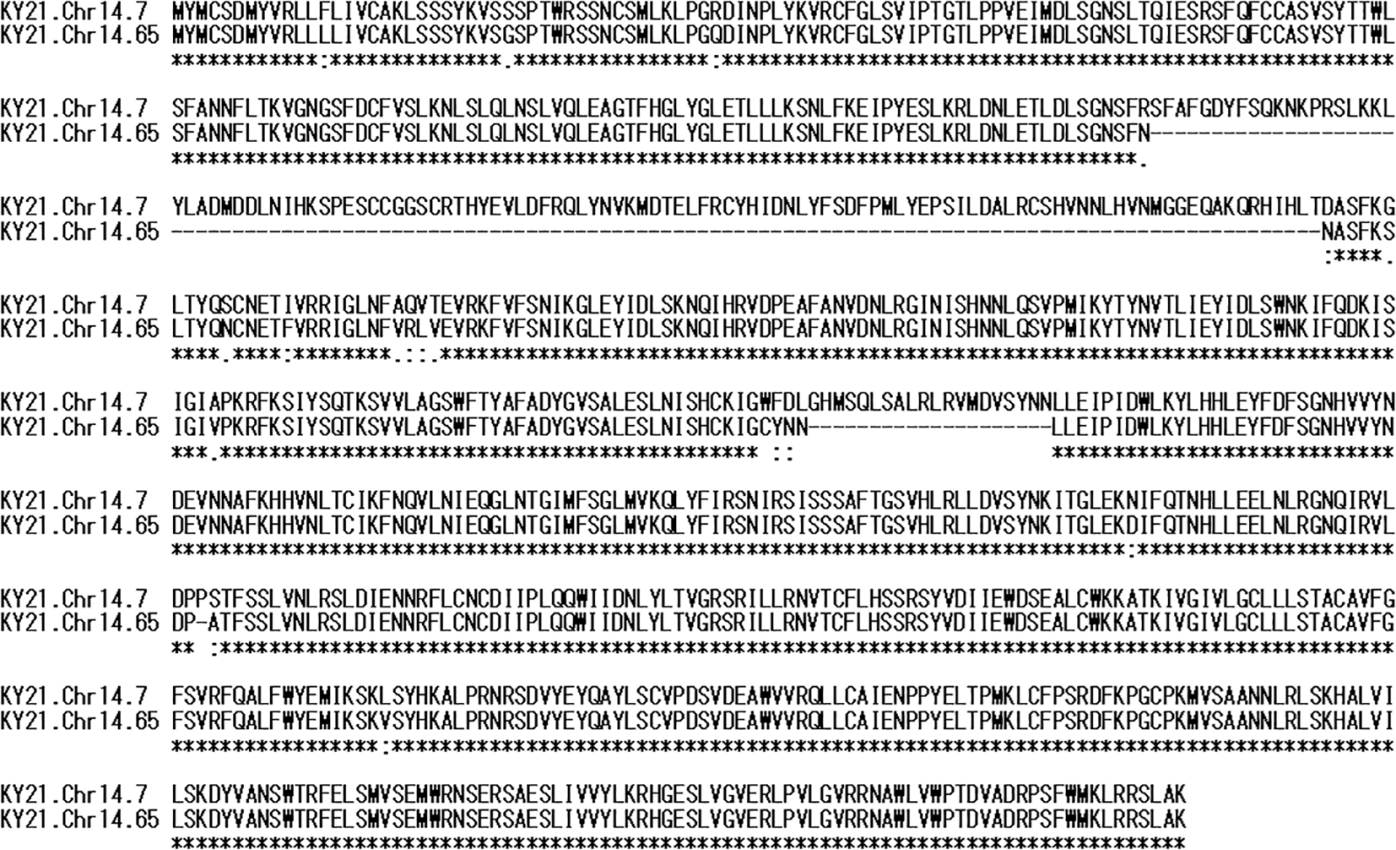
Amino acid sequences of CiTLR1 and CiTLRs1. Sequence alignment of KY21.Chr14.7. (CiTLR1 and -13) and KY21.Chr14.7 (CiTLRs1).

**Figure 3:**
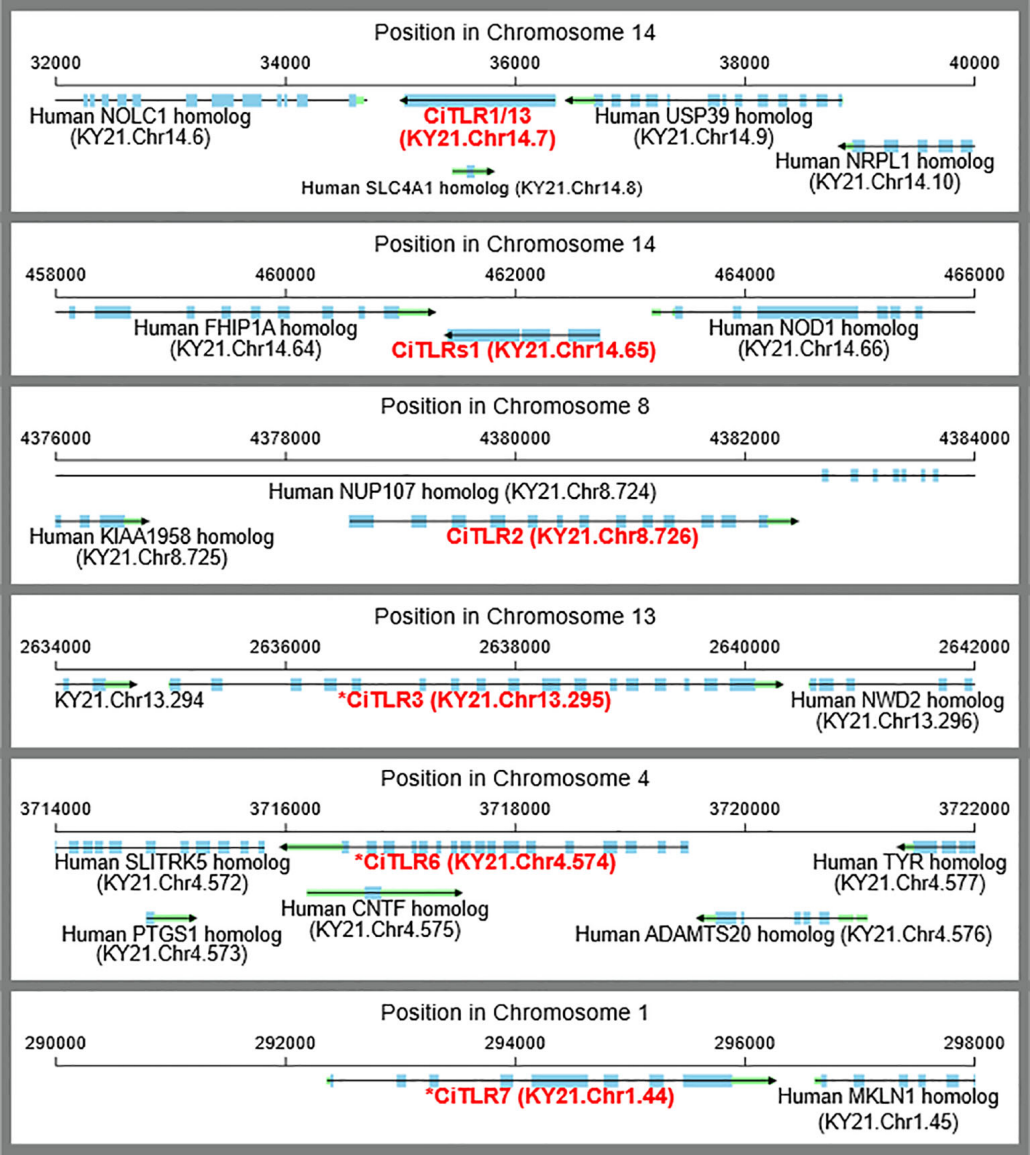
Gene structures and chromosomal loci of CiTLR genes. Gene structures, and chromosomal locus of CiTLRs. Blue and green bars indicate exons and UTRs, respectively.

A short protein homologous to *TLR1* (KY21.Chr14.65) was detected with low sequence coverage (47.42%) (**Table 1**). To investigate the cause of this low coverage, we aligned the amino acid sequences of KY21.Chr14.7 and KY21.Chr14.65, which revealed two gaps totaling 134 amino acids (**Figure 2**). The remaining aligned regions showed a high identity of 97.5% (731/749). Both KY21.Chr14.7 and KY21.Chr14.65 are located on chromosome 14, approximately 42 kb apart, while other TLRs were located on different chromosomes (**Figure 3**). Furthermore KY21.Chr14.7 is a transcript consisting of a single exon, while KY21.Chr14.7 and other TLRs are presumed to be divided into three exons encoding a short protein. These results indicate that KY21.Chr14.65 is a different gene from KY21.Chr14.7.

### 3.2 Evaluation of domain structures

**Figure 4:**
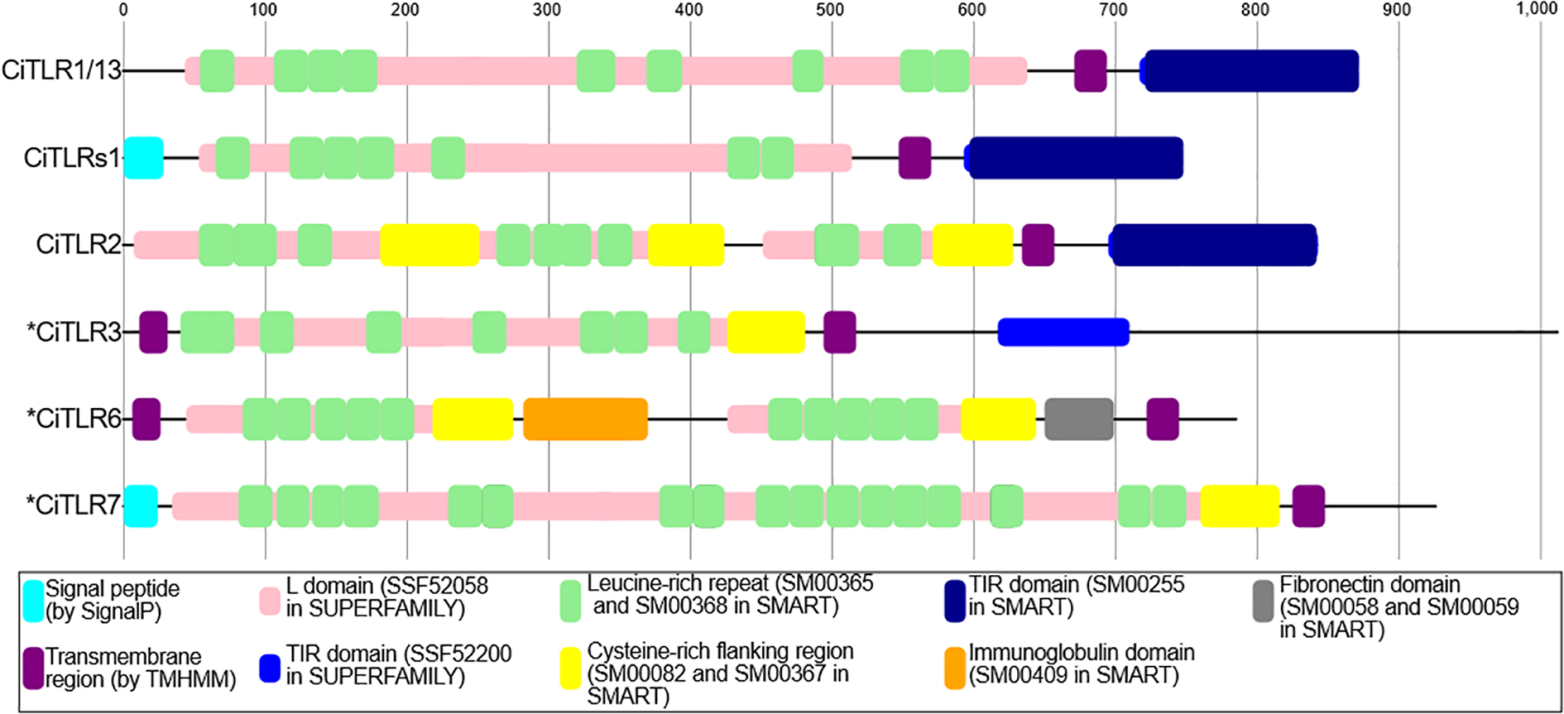
Structural organization of *Ciona* TLRs and TLR-like proteins. The functional domains predicted by TMHMM, SignalP, SUPERFAMILY, and SMART analyses. * indicates CiTLR-like genes that lack a complete TIR domain. Gene annotations were based on the information provided in the NCBI Genbank.

To investigate whether these putative *Ciona* TLRs possess the signature or structural features of TLR, we subsequently reanalyzed the domain structures of all blast-detected *Ciona* TLRs using TMHMM, SignalP, SUPERFAMILY, PANTHER, and SMART as implemented in InterProScan version 6 (Blum et al., 2025). As shown in **Figure 4**, all six putative TLR sequences exhibited multiple LRRs and transmembrane domains. However, the essential intracellular TIR domain was identified only in CiTLR1, CiTLR2, and CiTLRs1 (**Figure 4**). Furthermore, PANTHER search predicted CiTLR3, 6, and 7 as non-TLR gene families (**Table 2**). Based on the above results, we redefined KY21.Chr14.7, KY21.Chr14.65, and KY21.Chr8.726 as *CiTLR1, -s1*, and -*2*, respectively. Furthermore, KY21.Chr13.295, KY21.Chr4.574, and KY21.Chr1.44, which lack a complete TIR domain, are denoted as *CiTLR3, *CiTLR6, and *CiTLR7, respectively. In addition, *CiTLRs1* is predicted to encode multiple LRRs, a transmembrane domain, and a TIR domain, all of which are essential features of TLRs. These structural characteristics indicate that *CiTLRs1* (KY21.Chr14.65) represents a novel, *bona fide Ciona* TLR that signals through the intracellular adaptor, *Myd88*.

**Table 2:** Protein family predicted by PANTHER.

Query	Panther ID	PANTHER name	Start	End	P-value
CiTLR1(and -13)	PTHR24365	TOLL-LIKE RECEPTOR	42	870	3.3E-77
CiTLRs1	PTHR24365	TOLL-LIKE RECEPTOR	42	870	3.3E-77
CiTLR2	PTHR24365	TOLL-LIKE RECEPTOR	330	843	7.3E-91
*CiTLR3	PTHR24365	TOLL-LIKE RECEPTOR	349	710	3.5E-70
*CiTLR6	PTHR24369	ANTIGEN BSP, PUTATIVE-RELATED	396	639	4E-90
*CiTLR7	PTHR24373	SLIT RELATED LEUCINE-RICH REPEAT NEURONAL PROTEIN	570	832	9.3E-114

On the other hand, *CiTLR3 was predicted to possess an incomplete TIR domain; while SUPERFAMILY implied its presence, other analyses failed to detect it explicitly. No TIR domains were detected in *CiTLR6 or *CiTLR7 by any of the domain prediction tools. Additionally, *CiTLR1* and *CiTLRs1* displayed nearly identical domain architectures (**Figure 4**). These results suggest that *CiTLR6 and *CiTLR7 are not canonical TLRs in an organism lacking a Myd88-independent signaling pathway for these putative TLR-like genes lacking TIR domains (**Supplementary Figure 1**, **Supplementary Table 1**).

Furthermore, we performed a molecular phylogenetic analysis. To obtain gene sets suitable for this analysis, which required genes with sufficient homology for multiple sequence alignment, we clustered genes from 10 vertebrate species, 3 tunicate species, 1 cephalochordate species, 1 hemichordate species, and 4 echinoderm species using OrthoFinder (Emms and Kelly, 2019) (**Supplementary Table 2**). OrthoFinder is a program that identifies homologous gene sets (orthogroups) across multiple species. OrthoFinder-based clustering and following molecular phylogenetic analysis of the LRR domain sequences demonstrated that *CiTLR1, s1* and *CiTLR7 were clustered into a single orthogroup together with vertebrate TLRs (**Supplementary Figure 2A**). Furthermore, *CiTLR2* formed a sister group to vertebrate TLR2s. Unexpectedly, *CiTLR1* and *s1* formed a clade with deuterostome-invertebrate TLRs, whereas *CiTLR7, which lacks a TIR domain, was positioned adjacent to the vertebrate *TLR5* clade and did not cluster with other tunicates, cephalochordates, or invertebrate deuterostomes. *CiTLR6* was classified into an orthogroup containing Slit guidance ligand (SLIT) and neurotrophic receptor tyrosine kinase (NTRK)-like proteins (**Supplementary Figure 2B**) which are different transmembrane proteins from TLRs (Aruga and Mikoshiba, 2003). Furthermore, *CiTLR3 clustered with a non-TLR protein of *Styela clava* (**Supplementary Figure 2C**).

Subsequently, we examined synteny conservation across representative species (**Supplementary Figure 3**). Human and zebrafish *TLR3* loci were both flanked by homologous genes, indicating clear conservation of synteny. Similarly, zebrafish *TLR8A* and human *TLR7/8* shared common neighboring genes on their respective chromosomes. In contrast, no conserved syntenic relationships were detected around the TLR loci in ascidians, which is consistent with frequent disruption of syntenic blocks in the *Ciona* genome (Berna and Alvarez-Valin, 2014).

To further evaluate their structural validity, we predicted the tertiary structures of the CiTLRs using AlphaFold3 (Abramson et al., 2024). As shown in **Supplementary Figure 4**, CiTLR1/13 displayed canonical TLR features, including a folded solenoid structure and a transmembrane helix. The LRR domain of *CiTLR2* displayed three-divided short-solenoid structures with cysteine-rich region, giving it a shape that differs from a typical solenoid structure. However, *CiTLR2* displayed a transmembrane helix and TIR domain, suggesting that the LRR domain take a typical solenoid structure upon pathogen binding. Motif searches revealed that CiTLR1 and CiTLRs1 harbor nine and seven LRR domains, respectively (**Figure 4**). Consistent with this result, the predicted structure of *CiTLRs1* was shorter than that of *CiTLR1* but showed a typical solenoid structure (**Supplementary Figure 4**). This also indicates that *CiTLRs1* is an authentic TLR.

Furthermore, *CiTLR1*, *CiTLR2* and *CiTLRs1* also displayed the representative TIR domain, which was composed of five parallel β-strands (βA–βE) surrounded by five α-helices (αA-αE), which are structural prerequisites for signal transduction by the TIR domains (Xu et al., 2000) (see hTLR4 in **Supplementary Figure 4**). In contrast, AlphaFold3 failed to confidently predict a structured TIR domain in *CiTLR3. Taken together, these structural analyses indicate that only *CiTLR1*, *CiTLR2*, and *CiTLRs1* possess domain compositions and structural features consistent with typical TLRs. Altogether, these protein organization, molecular phylogenetic, genomic organization, and structural analyses indicate that *CiTLR3, *CiTLR6, and *CiTLR7 are not canonical TLRs.

### 3.3 Tissue distribution of CiTLRs

**Figure 5:**
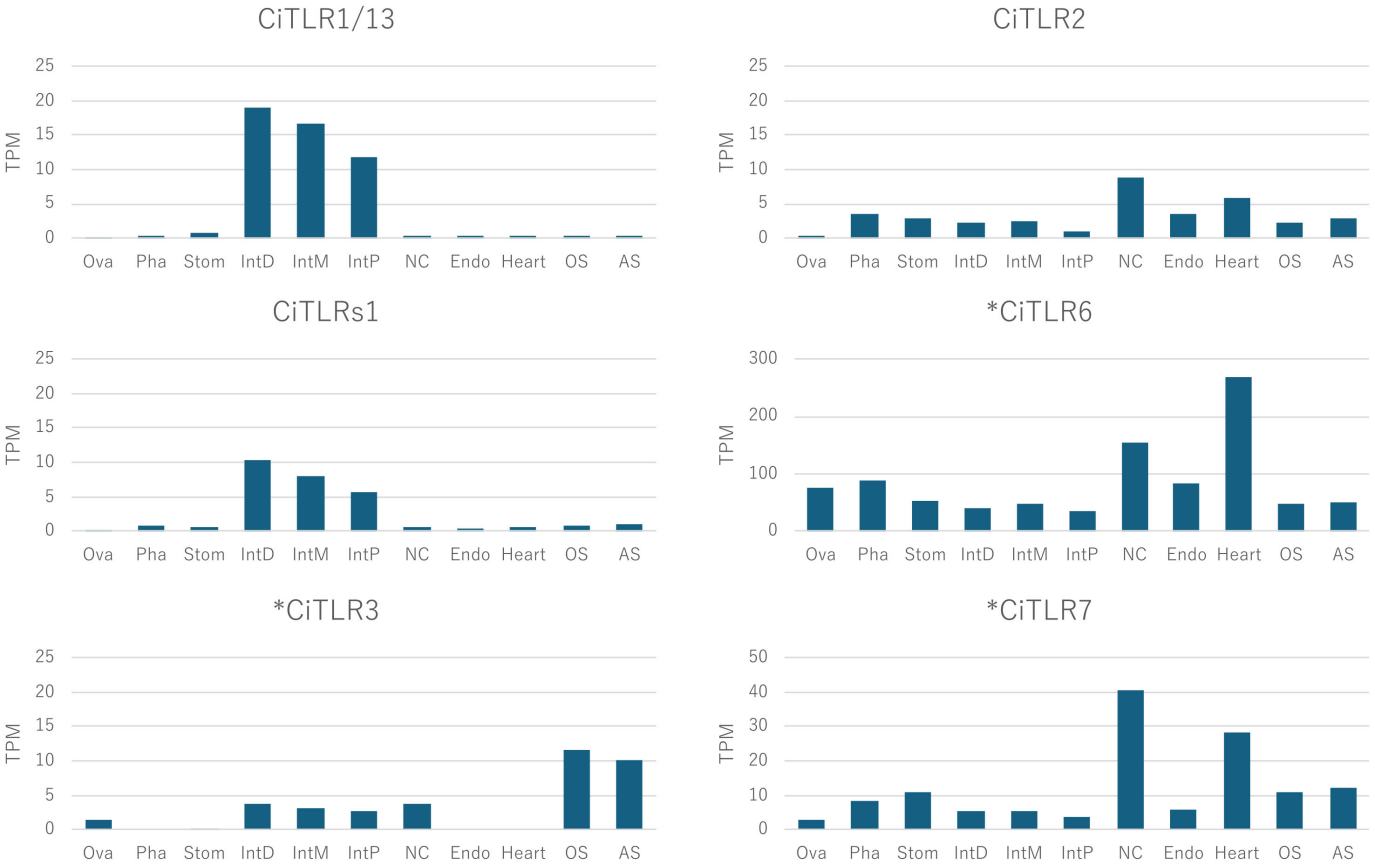
Tissue distribution of CiTLR and CiTLR-like mRNA in adult *Ciona*. Each gene expression was evaluated using previous RNA-seq data. Ova, ovary; Pha, pharynx; Stom, stomach; IntD, distal intestine; IntM, middle intestine; IntP, proximal intestine; NC, neural complex; Endo, endostyle; OS, oral siphon; AS, atrial siphon. * Indicates CiTLR-like genes that lack a complete TIR domain.

To investigate the expression profiles of *Ciona* TLRs (*CiTLR1*, *-2*, and *-s1*) and TLR-like genes (*CiTLR3, 6, 7), we reanalyzed published *Ciona* transcriptome datasets (Matsubara et al., 2021). The transcriptome analysis demonstrated the distinct expression profiles (**Figure 5**). While *CiTLR1*and *s1* were predominantly expressed in the intestine, the expression of *CiTLR2, -6*, and -*7* was detected across various tissues.

### 3.4 scRNA-seq of TLRs in *Ciona* hemocytes

**Figure 6:**
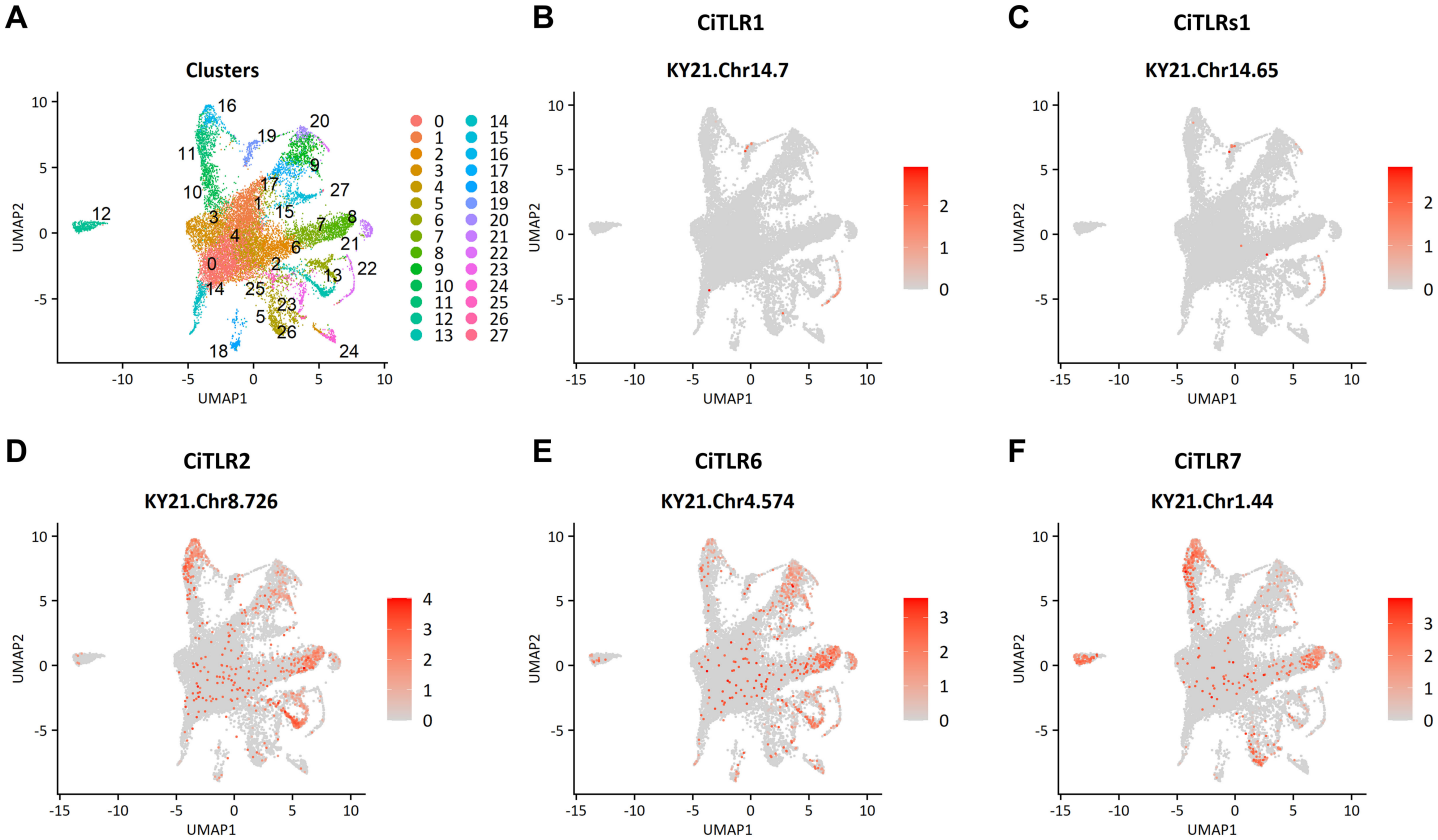
scRNA-seq analysis of CiTLR expression in *Ciona* hemocytes. **(A)** UMAP visualization of scRNA-seq data showing Seurat-defined clusters. **(B–F)** Expression of CiTLR genes.

**Figure 7:**
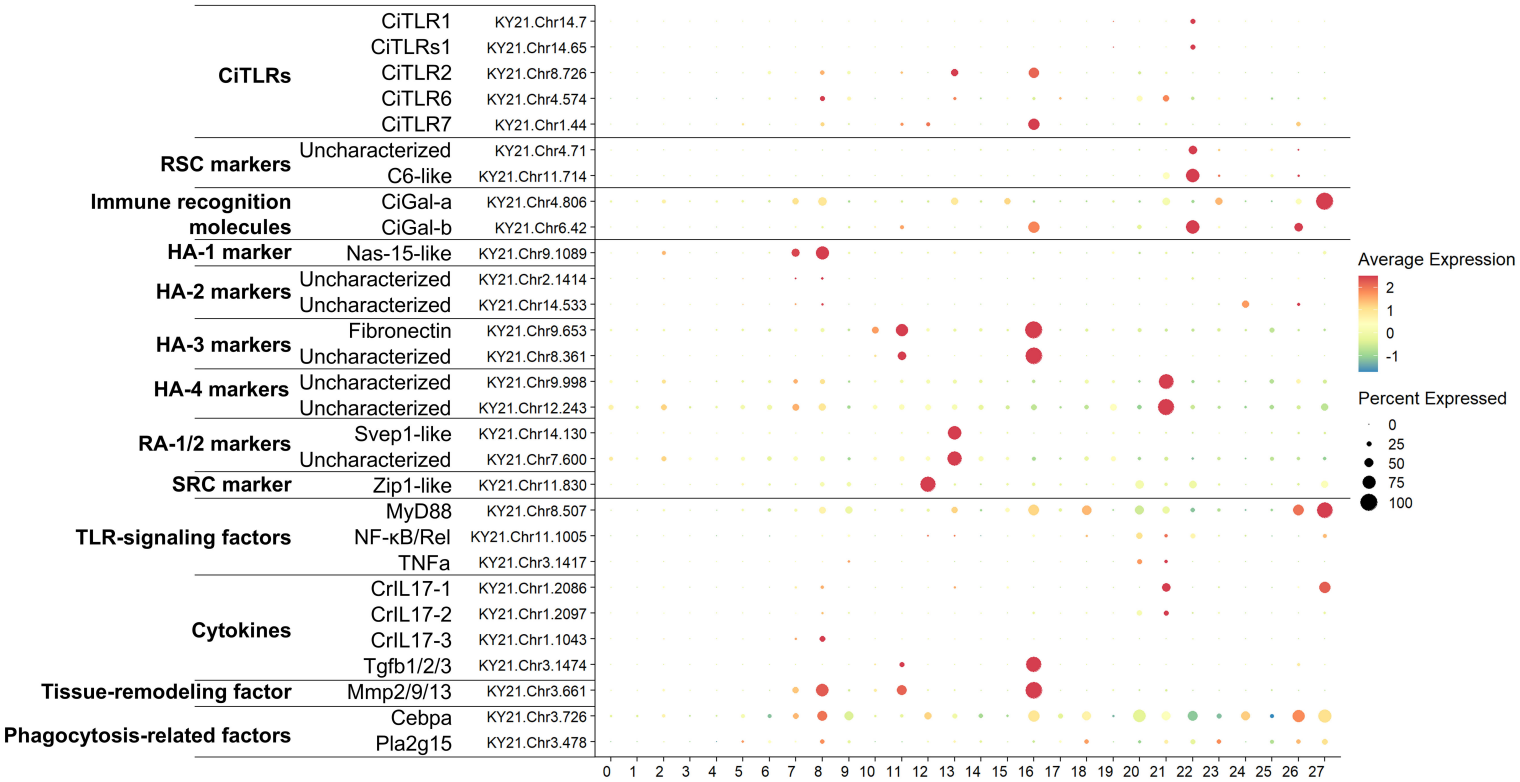
Expression patterns (Dot plot) of CiTLR genes in *Ciona* hemocytes. Dot plot summary showing the expression levels (color) and the proportion of expressing cells (dot size) for selected genes. Cluster numbers correspond to those shown in **Figures 6**.

To investigate the expression profiles of *CiTLR* genes in hemocytes, we performed scRNA-seq analyses (accession number: PRJNA1308730) and found that *Ciona* hemocytes were divided into 28 distinct clusters based on gene expression profiles (**Figures 6** and **7**). We then referred to and assigned each cluster to recently revised morphotypes of *Ciona* hemocytes (Scully et al., 2025), and the results are summarized in **Table 3**. *CiTLR1* and *CiTLRs1* were specifically expressed in cluster 22 (**Figure 6**), which was characterized as round spreading cells (RSCs) (**Table 3**; Scully et al., 2025). This cluster also expressed the complement *C6-like* gene (an RSC marker gene, KY21.Chr11.714) and the *CiGal-b* gene (KY21.Chr6.42) (**Figure 6**; Scully et al., 2025). These results indicate that *CiTLR1* and *CiTLRs1* are expressed predominantly in RSCs. *CiTLR3 expression was not detected in any cluster. *CiTLR2*, *CiTLR6, and *CiTLR7 were predominantly expressed either or both in clusters 8 and 16, which are characterized by the expression of the HA marker genes, zinc metalloproteinase *Nas-15-like* (KY21.Chr9.1089) and *fibronectin* (KY21.Chr9.653) (**Figures 6** and **7**). The TLR-signaling factor *MyD88* was also expressed in clusters 8 and 16, while *NF-κB/Rel* and *TNF*α were mainly expressed in cluster 21 with *CiTLR7 and *CrIL17-1* (**Figure 7**). Moreover, several known immune-related genes including *CrIL17* (Vizzini et al., 2015), *Tgfb1/2/3* (Vizzini et al., 2016), *Mmp2/9/13* (Cancemi et al., 2019), and phagocytosis-related factors (*Cebpa*, and *Pla2g15)* (Nagahata et al., 2022) were expressed in clusters 8 and 16 (**Figure 7**). *CiTLR2* and *CiTLR6 were also expressed in cluster 13, which is characterized by the expression of the marker gene of refractile amoebocytes (RAs) (*Svep1-like*, KY21.Chr14.130) (**Figure 7**; Scully et al., 2025). *CiTLR7 was also expressed in cluster 12, which is characterized by the marker gene of signet ring cells (SRCs) (*Zip1-like*, KY21.Chr11.830) (**Figure 7**; Scully et al., 2025). These results indicate that *CiTLR2*, *CiTLR6, and *CiTLR7 are most abundantly expressed in HA family cells, with lower expression in RA-1/2, and in SRCs (only *CiTLR7). A summary of *CiTLR* gene expression is shown in **Table 4**.

**Table 3:** Correspondence between cell morphotypes and cluster numbers.

Former morphotypes	Revised morphotypes (Scully et al., 2025)	Transcriptional classification (Scully et al., 2025)	Cluster number (Scully et al., 2025)	Cluster number in the current study
LLC	HLC	cMPP	0, 14, 1	26, 15, 20
	RC	cLRP-1	1, 7	9, 17, 20
		pre-RA	4	6
		RC	21	23, 25
	RSC	RSC	10	22
HA	HA	HA-1	3	8, 7
		HA-2	9	24, 26
		HA-3	13	16, 11
		HA-4	15	21
		HA-5	31	11, 10
	BLC	BLC	11	27, 15, 24
GH	GA	GA	12	26, 5
	SGH	SGH-1	24	24, 3
		SGH-2	29	17
GH/MC	LGH/MC	LGH/MC	16	23
SRC	SRC	SRC	22	12
URG	URG	URG-1	19	14
	URG, ND	URG-2 and ND-2	27, 23	18, 5
CC/URG	URG	URG-3	28	ND
CC	ICC	ICC	18	25
RA	RA	RA-1 and -2	8, 25	13

BLC, Blebbing-like cell; CC, Compartment cell; cMPP, Candidate multipotent progenitor; cLRP-1, Candidate lineage-restricted progenitor 1;GA, Granular amoebocyte; GH, Granular hemocyte; HA, Hyaline amoebocyte; HLC, Hemoblast-like cell; ICC, Irregular compartment cell; LGH Large granules hemocyte; LLC, Lymphocyte-like cell; MC, Morula cell; ND, Not determined; pre-RA, Pre-refractile amoebocyte; RA, Refractile amoebocyte; RC, Round cell; RSC, Round spreading cell; SGH, Small granules hemocyte; SRC, Signet ring cell; URG, Unilocular refractile granulocyte

**Table 4:** Summary of CiTLR gene expression in *Ciona* hemocytes.

Gene name	Accession ID	Clusters expressed in	Morphotypes	Co-expression with
CiTLR1	KY21.Chr14.7	22	RSC	C6-like and CiGal-b
CiTLRs1	KY21.Chr14.65	22	RSC	C6-like and CiGal-b
CiTLR2	KY21.Chr8.726	8, 13, 16	HA, RA	MyD88, Cebpa, Pla2g15, CrILs, Tgfb1/2/3 and Mmp2/9/13
*CiTLR3	KY21.Chr13.295	ND	ND	ND
*CiTLR6	KY21.Chr4.574	8, 13, 21	HA, RA	MyD88, Cebpa, Pla2g15, NF-κB/Rel, TNFα, CrIL17-1, and Mmp2/9/13
*CiTLR7	KY21.Chr1.44	8, 11, 12, 16, 26	HA, SRC, RA	MyD88, Cebpa, Pla2g15, CrILs, Tgfb1/2/3 and Mmp2/9/13

HA, Hyaline amoebocyte; ND, Not determined; RA, Refractile amoebocyte; RSC, Round spreading cell; SRC, Signet ring cell

## 4 Discussion

To date, six *Ciona* TLRs, TLR1, -2, -3, -6, -7, and -13 have been annotated on the NCBI genome database (GCF_000224145.3). However, the present study, through in-depth genomic, domain organization, and three-dimensional structural analyses, revealed that TLR13 is identical to TLR1 and that *CiTLR3, -6, or -7 are not typical TLRs due to the absence of a TIR domain, which is critical for TLR signal transduction. This overestimation of TLR numbers likely stems from inaccurate annotations based on early BLAST-based analyses of the initial *Ciona* genome, without adequate consideration of domain architecture. Notably, these misannotations have remained uncorrected for over twenty years. In addition, the apparent duplication of TLR1 and TLR13 is likely the result of either genome assembly error or annotation artifacts. It is also noteworthy that gene structure localization, specific interaction with PAMPs, and downstream signaling of CiTLR1 and CiTLR2 have been experimentally demonstrated (Sasaki et al., 2009; Satake and Sasaki, 2010; Nonaka and Satake, 2010; Satake and Sekiguchi, 2012; Satake et al., 2019), whereas no such evidence has been reported for *CiTLR3, -6, -7, or -13. Thus, the identification of authentic TLRs requires gene expression data and functional demonstration of PAMP binding and signal transduction, but should not be based merely on simple sequence comparison. Similar risks of misannotation may also exist in other proteins in any animal species.

It may be noteworthy that several “soluble” or “decoy” TLRs that lack both a transmembrane region and a TIR domain have been identified in vertebrates and modulate innate immune responses (Tsujita et al., 2004; Park et al., 2008; Zunt et al., 2009; Henrick et al., 2016; Fukui et al., 2018; Zhang et al., 2023; Houssen et al., 2024). Most of these, unlike *CiTLR3, *CiTLR6, or *CiTLR7, arise from full-length TLRs via ectodomain proteolysis (Zunt et al., 2009; Park et al., 2008; Henrick et al., 2016; Fukui et al., 2018; Houssen et al., 2024). In contrast, the soluble TLR5s (sTLR5) of teleosts and lamprey (*Petromyzon marinus*) are encoded by a gene distinct from the canonical membrane-type TLR5 (TLR5M) (Tsujita et al., 2004; Zhang et al., 2023; Liao et al., 2024), which is reminiscent of the *CiTLR3, *CiTLR6, and *CiTLR7 genes (**Figure 4**). In particular, molecular phylogenetic trees (**Supplementary Figure 2**) indicate that *CiTLR7, unlike *CiTLR3 or *CiTLR6, falls within the vertebrate TLR clade. The phylogenetic relatedness of sTLR5 to TLR5 supports the idea that sTLR5 evolved via gene duplication of TLR5 in teleost-specific lineage (Zhang et al., 2023). Likewise, the present molecular phylogenetic tree (**Supplementary Figure 2A**) demonstrated that the *P. marinus* membrane TLR5 (PmTLR5) and soluble TLR5 (PmTLR5S) were generated in the *Petromyzon*-specific lineage. However, the present analyses provide no phylogenetic evidence that *CiTLR7 was generated by duplication of other *CiTLR* genes (**Supplementary Figure 2A**). Consequently, it remains unclear whether *CiTLR7* can function as a *Ciona*-specific soluble TLR. Collectively, the designations “*CiTLR3, -6, and -7” are at present not appropriate and should preferably be replaced by “CiLRR-containing protein-1, -2, and -3” or other nomenclature reflecting their biological functions.

In keeping with these issues, incorrect identification of TLRs originated from rough immunohistochemical studies. Several studies (Lauriano et al., 2021; Alesci et al., 2022; Bisanti et al., 2024) demonstrated the presence of “TLR2” or “TLR4” in *Ciona* and other ascidians by immunohistochemical analyses using antibodies against human TLR2 or TLR4, respectively, but without molecular characterization of the cognate TLRs. These studies failed to take into account that homologs of human TLRs are not necessarily conserved in ascidians and that antibodies raised against human TLRs may recognize different targets in non-human species. Indeed, CiTLR1 and -2 exhibited “hybrid PAMP recognition” of human TLRs but no responses to LPS, as is recognized by the TLR4-MD2 complex in mammals (Takeda and Akira, 2015; Sasaki et al., 2009; Satake and Sekiguchi, 2012; Satake et al., 2019). In addition, no ortholog of MD2 has been identified in *Ciona*, whereas essential factors for TLR signaling such as MyD88 and NF-κB are conserved in *Ciona* (Azumi et al., 2003; Sasaki et al., 2009; Satake and Sasaki, 2010; Satake and Sekiguchi, 2012; Satake et al., 2019; Longo et al., 2021; Liberti et al., 2023) (**Supplementary Figure 1**). Therefore, the evolutionary conservation of *Ciona* homolog of human TLR4 and the direct recognition of LPS by TLRs in *Ciona* remains highly questionable, although CiTLRs are likely to be involved in immune responses to LPS (Arizza et al., 2020; Longo et al., 2021).

LPS exposure is known to trigger various inflammatory and/or immune responses in *Ciona* (Nonaka and Satake, 2010; Arizza et al., 2020; Longo et al., 2021; Vizzini et al., 2021; La Paglia et al., 2023; Dumas et al., 2023; Liberti et al., 2023; Matsubara et al., 2024; Bisanti et al., 2024). Nevertheless, as stated above, CiTLRs cannot recognize LPS (Sasaki et al., 2009; Satake and Sasaki, 2010; Satake and Sekiguchi, 2012), suggesting other immune pathways are responsible for mediating responses to LPS in *Ciona*. Interestingly, Arizza et al. demonstrated that CiTLR1 and CiTLR2 function in distinct phases of the LPS-induced immune response. CiTLR1 is rapidly upregulated during the early response phase and is likely involved in the immediate activation of pro-inflammatory signaling (Arizza et al., 2020). In contrast, CiTLR2 shows a biphasic expression pattern and is associated with sustained immune modulation, including transcriptional activation of downstream effectors in the later phase (Arizza et al., 2020). These findings suggest distinct immune roles for the two TLRs. Combined with these findings, the expression of the *CiTLR3, -6, and -7 genes (**Figure 5**) suggests that these truncated TLR-like proteins also play some roles in pathogen recognition likely through their LRR domains, although they do not function as authentic TLRs.

Also of significance in the present study is the identification of a novel putative *Ciona* TLR gene, CiTLRs1 (**Figures 2** and 4). Although its full-length sequence is shorter than that of CiTLR1 and CiTLR2, CiTLRs1 harbors a complete set of structural domains, including the LRR, transmembrane domain, and TIR domain. These features suggest that CiTLRs1 is an authentic *Ciona* TLR. Additionally, the similarity in gene expression profiles between CiTLRs1 and CiTLR1 (**Figure 5**) suggests that CiTLRs1 may be expressed in the same immune-responsive cell type, potentially recognizing distinct PAMPs. The precise localization and immune function of CiTLRs1 awaits further studies.

*Ciona* hemocytes were previously classified into 7 types (Longo et al., 2021) and recently redefined into 13 morphotypes and 26–33 transcriptionally distinct cellular states identified by scRNA-seq (Scully et al., 2025). Our independent scRNA-seq dataset was generally consistent with this and each cluster could be assigned to corresponding morphotypes (**Table 3**). We demonstrated that *CiTLR1* and *CiTLRs1* were expressed predominantly in RSC, and *CiTLR2*, *CiTLR6, and *CiTLR7 were expressed most abundantly in HA family cells (**Figures 6**, **7**, **Table 4**). RSCs have been considered to possess hematopoietic and/or stem cell-like activities. However, our scRNA-seq data, showing the specific expression of *CiTLR1* and *CiTLRs1*, along with the expression of *C6-like* and *CiGal-b*, suggest that RSCs function in PAMP recognition and immune responses (**Figures 6**, 7, and **Table 4**). HAs are motile agranular hemocytes with strong phagocytic activity, and were found to migrate to infection or injury sites, engulf pathogens and debris, and contribute to innate immune defense and tissue repair (Longo et al., 2021). These findings are supported by our data showing the expression of CiTLR2, *CiTLR6, *CiTLR7, phagocytosis-related factors (*Cebpa* and *Pla2g15*), inflammation signaling and tissue-remodeling factors (*CrILs*, *Tgfb1/2/3* and *Mmp2/9/13*) in HA family cells (**Figures 6**, 7, and **Table 4**). Combined with these findings, the present expression profiles in hemocytes suggest that these CiTLRs (CiTLR1, 2, and s1) and their related proteins (*CiTLR6 and -7) are likely to play distinct immune roles in *Ciona*.

In conclusion, the present study substantiated that only CiTLR1 and CiTLR2 are regarded as *bona fide* Toll-like receptors, and detected another putative CiTLR, CiTLRs1. The structural criteria of TLRs, the classification and total number must be critically revised as demonstrated in this study.

## Data Availability

The datasets presented in this study can be found in online repositories. The names of the repository/repositories and accession number(s) can be found in the article/**Supplementary Material**.
