# Supplementary material for: Redefinition of the toll-like receptor repertoire in Ciona robusta through genomic, structural, and expression analyses
Source: Front Cell Infect Microbiol. 2026 Jan 6;15:1716256. doi: 10.3389/fcimb.2025.1716256 (PMC12816324; doi:10.3389/fcimb.2025.1716256)
Supplement: Supplementary file 1 [file DataSheet1.docx]

Supplementary Material

**Supplementary Table 1.**  **TLR pathway-related genes in *Ciona*.**

| Product | description | human protein (query) | Ciona protein (Blast hit) | e-value |
| --- | --- | --- | --- | --- |
| MyD88 | Myeloid differentiation primary response protein MyD88 | MYD88_HUMAN | KY21.Chr8.507.v1.SL1-1 | 4.66E-33 |
| IRAK4 | Interleukin-1 receptor-associated kinase 4 | IRAK4_HUMAN | KY21.Chr8.1013.v2.SL1-1 | 4.05E-74 |
| TOLLIP | Toll-interacting protein | TOLIP_HUMAN | KY21.Chr5.430.v1.SL2-1 | 1.11E-60 |
| IRAKM | Interleukin-1 receptor-associated kinase 3 | IRAK3_HUMAN | No ortholog | |
| IRAK1 | Interleukin-1 receptor-associated kinase 1 | IRAK1_HUMAN | KY21.Chr1.1173.v1.ND1-1 | 1.13E-55 |
| IRAK2 | Interleukin-1 receptor-associated kinase 2 | IRAK2_HUMAN | No ortholog | |
| TRAF6 | TNF receptor-associated factor 6 | TRAF6_HUMAN | KY21.Chr4.226.v1.nonSL4-1 | 1.30E-50 |
| ECSIT | Evolutionarily conserved signaling intermediate in Toll pathway, mitochondrial | ECSIT_HUMAN | KY21.Chr8.679.v1.nonSL1-1 | 7.19E-35 |
| Ubc13 | Ubiquitin-conjugating enzyme E2 N | UBE2N_HUMAN | KY21.Chr5.576.v1.SL1-1 | 3.04E-87 |
| TAK1 | Nuclear receptor subfamily 2 group C member 2 | NR2C2_HUMAN | KY21.Chr8.532.v1.SL1-1 | 6.22E-138 |
| TAB1 | TGF-beta-activated kinase 1 and MAP3K7-binding protein 1 | TAB1_HUMAN | KY21.Chr10.27.v1.SL1-1 | 1.29E-108 |
| TAB2 | TGF-beta-activated kinase 1 and MAP3K7-binding protein 2 | TAB2_HUMAN | KY21.Chr6.370.v1.SL2-1 | 7.51E-27 |
| MKK4 | Dual specificity mitogen-activated protein kinase kinase 4 | MP2K4_HUMAN | KY21.Chr8.933.v1.SL1-1 | 3.55E-164 |
| MKK7 | Dual specificity mitogen-activated protein kinase kinase 7 | MP2K7_HUMAN | KY21.Chr9.522.v1.SL1-1 | 8.89E-164 |
| JNK | Mitogen-activated protein kinase 8 | MK08_HUMAN | KY21.Chr14.295.v1.nonSL2-1 | 0.00 |
| NF-κB | Nuclear factor NF-kappa-B | NFKB1_HUMAN | KY21.Chr7.418.v1.SL1-1 | 1.80E-127 |
| MKK3 | Dual specificity mitogen-activated protein kinase kinase 3 | MP2K3_HUMAN | KY21.Chr5.1118.v1.SL1-1 | 1.78E-144 |
| MKK6 | Dual specificity mitogen-activated protein kinase kinase 6 | MP2K6_HUMAN | KY21.Chr5.1118.v1.SL1-1 | 2.92E-146 |
| p38 MAPK | Mitogen-activated protein kinase 11 | MK11_HUMAN | KY21.Chr6.586.v1.SL2-4 | 0.00 |
| TRAF3 | TNF receptor-associated factor 3 | TRAF3_HUMAN | KY21.Chr10.43.v1.SL1-1 | 1.30E-103 |
| TBK1 | Serine/threonine-protein kinase TBK1 | TBK1_HUMAN | KY21.Chr8.1137.v2.SL2-1 | 6.40E-178 |
| IκBα | NF-kappa-B inhibitor alpha | IKBA_HUMAN | KY21.Chr4.275.v1.nonSL13-1 | 1.26E-33 |
| RelA | Transcription factor p65 | TF65_HUMAN | KY21.Chr11.1005.v1.SL1-1 | 7.10E-99 |
| CD14 | Monocyte differentiation antigen CD14 | CD14_HUMAN | No ortholog | |
| MD2 | Lymphocyte antigen 96 | LY96_HUMAN | No ortholog | |
| TIRAP | Toll/interleukin-1 receptor domain-containing adapter protein | TIRAP_HUMAN | No ortholog | |
| TCAM1 | TIR domain-containing adapter molecule 1 | TCAM1_HUMAN | No ortholog | |
| TCAM2 | TIR domain-containing adapter molecule 2 | TCAM2_HUMAN | No ortholog | |
| SOCS1 | Suppressor of cytokine signaling 1 | SOCS1_HUMAN | No ortholog | |
| SARM1 | NAD(+) hydrolase SARM1 | SARM1_HUMAN | No ortholog | |
| IRF3 | Interferon regulatory factor 3 | IRF3_HUMAN | No ortholog | |
| IRF7 | Interferon regulatory factor 7 | IRF7_HUMAN | No ortholog | |

**Supplementary Table 2. Genome sequences used for Orthofinder.**

| phylum | Organism Name | Assembly Accession (or URL) |
| --- | --- | --- |
| Vertebrates | Alligator mississippiensis | GCF_000281125.3 |
| Vertebrates | Carcharodon carcharias | GCF_017639515.1 |
| Vertebrates | Danio rerio | GCF_049306965.1 |
| Vertebrates | Emys orbicularis | GCF_028017835.1 |
| Vertebrates | Gallus gallus | GCF_016700215.2 |
| Vertebrates | Homo sapiens | GCF_000001405.40 |
| Vertebrates | Latimeria chalumnae | GCF_037176945.1 |
| Vertebrates | Myxine glutinosa | GCF_040869285.1 |
| Vertebrates | Petromyzon marinus | GCF_010993605.1 |
| Vertebrates | Xenopus tropicalis | GCF_000004195.4 |
| Tunicates | Ciona intestinalis | https://ghost.zool.kyoto-u.ac.jp/download_ht.html |
| Tunicates | Oikopleura dioica | GCA_907165135.1 |
| Tunicates | Styela clava | GCF_013122585.1 |
| cephalochordates | Branchiostoma floridae | GCF_000003815.2 |
| Hemichordates | Saccoglossus kowalevskii | GCF_000003605.2 |
| Echinoderms | Acanthaster planci | GCF_001949145.1 |
| Echinoderms | Antedon mediterranea | GCF_964355755.1 |
| Echinoderms | Apostichopus japonicus | GCF_037975245.1 |
| Echinoderms | Asterias amurensis | GCF_032118995.1 |


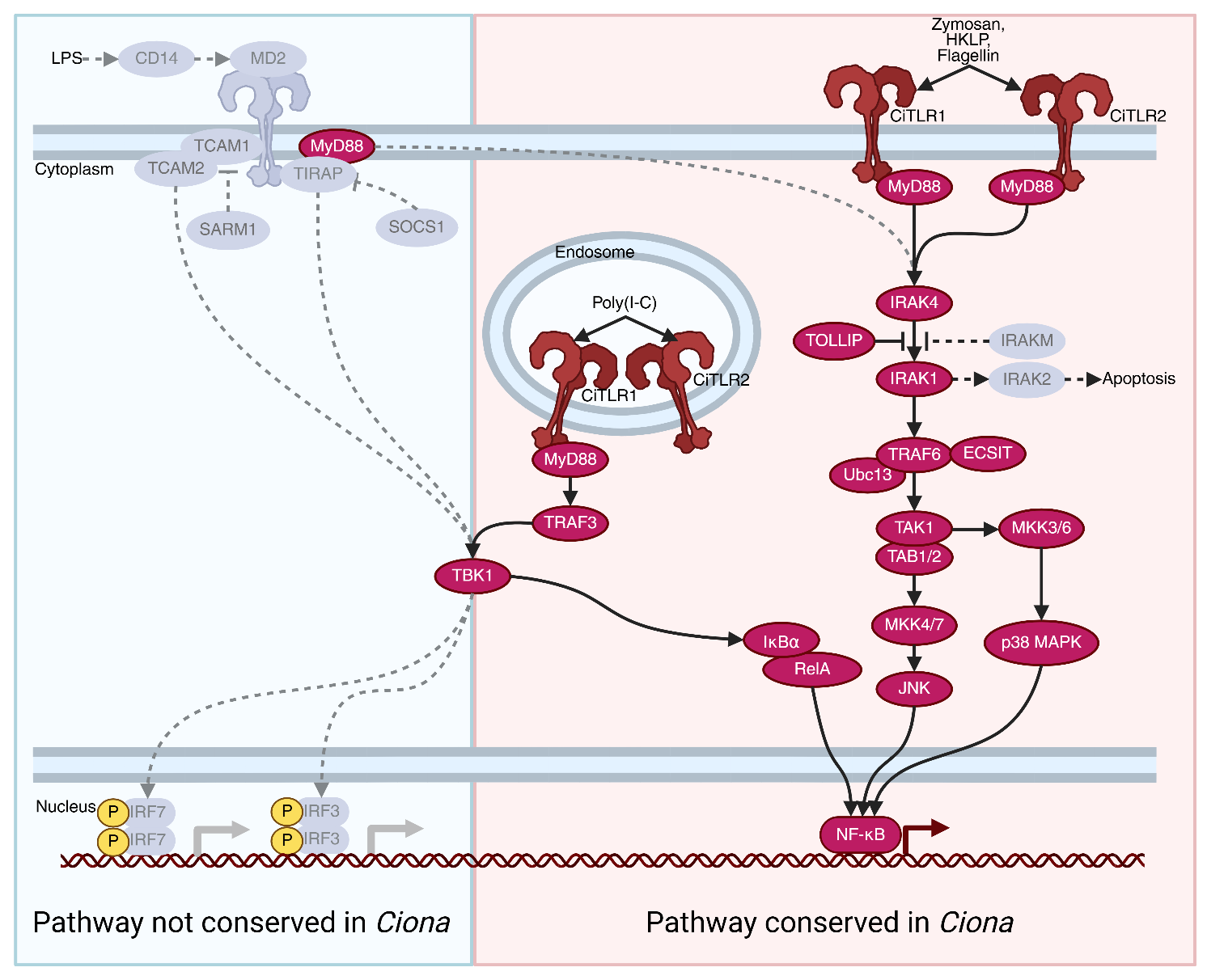


**Supplementary Figure 1.**  **TLR pathway-related genes in *Ciona*.**

Genes found in the HT genome with an e-value of 1e-20 or lower in a BLAST search using human genes as the query are shown in red, while those not found are shown in gray. The blast-detected genes were listed in supplementary Table 1. The figure was created in BioRender. Matsubara, S. (2025) <https://BioRender.com/pif9l9z> .

**
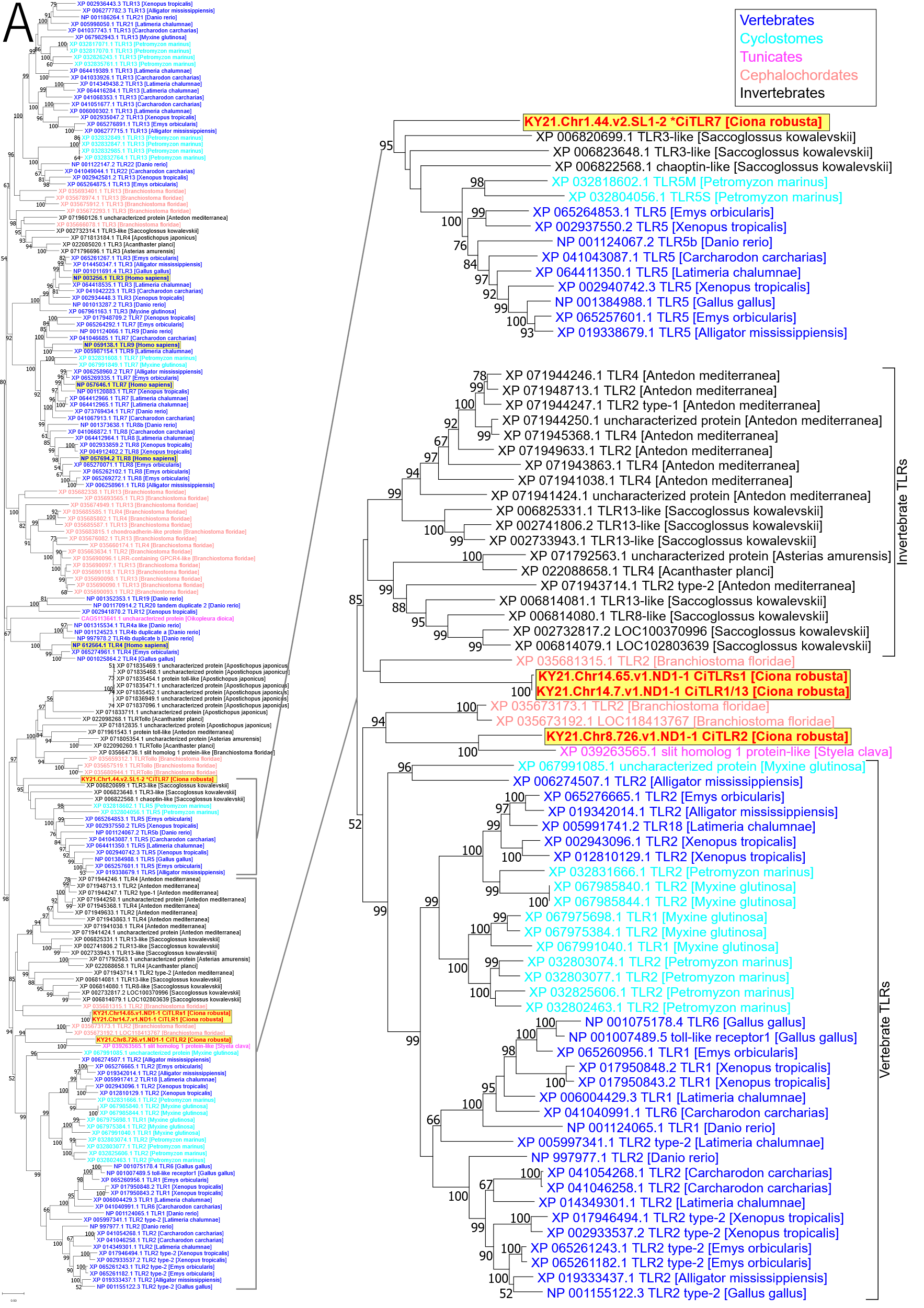

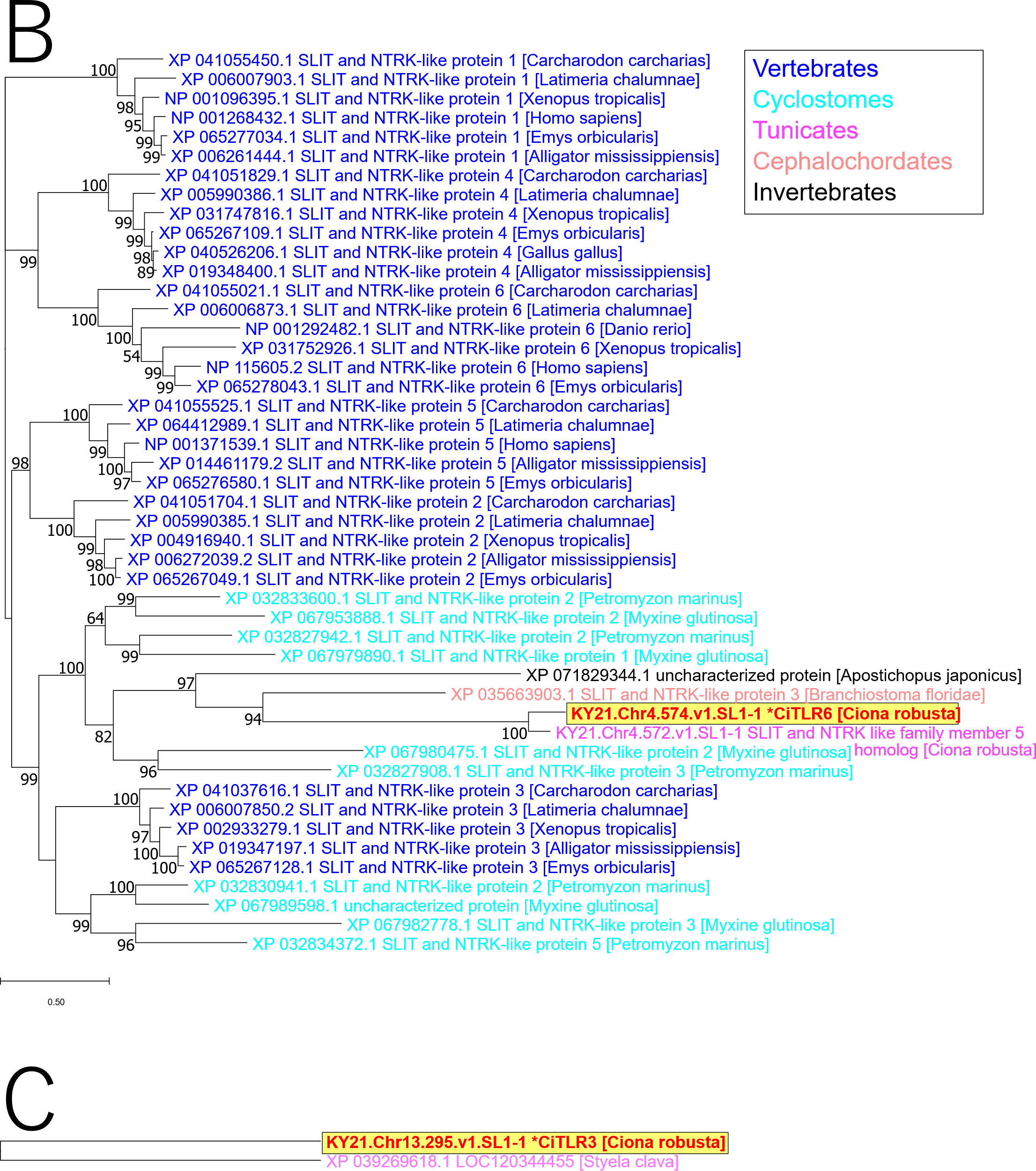
**

**Supplementary Figure 2.**  **Molecular phylogenetic tree of the orthogroup including CiTLRs.** Orthogroups CiTLR1/13, 1s, 2 and 7 (A), the orthogroup including *CiTLR6 (B) and the orthogroup including *CiTLR3. Each nodes were colored based on taxon species and CiTLRs and human TLRs were highlighted in yellow.

**
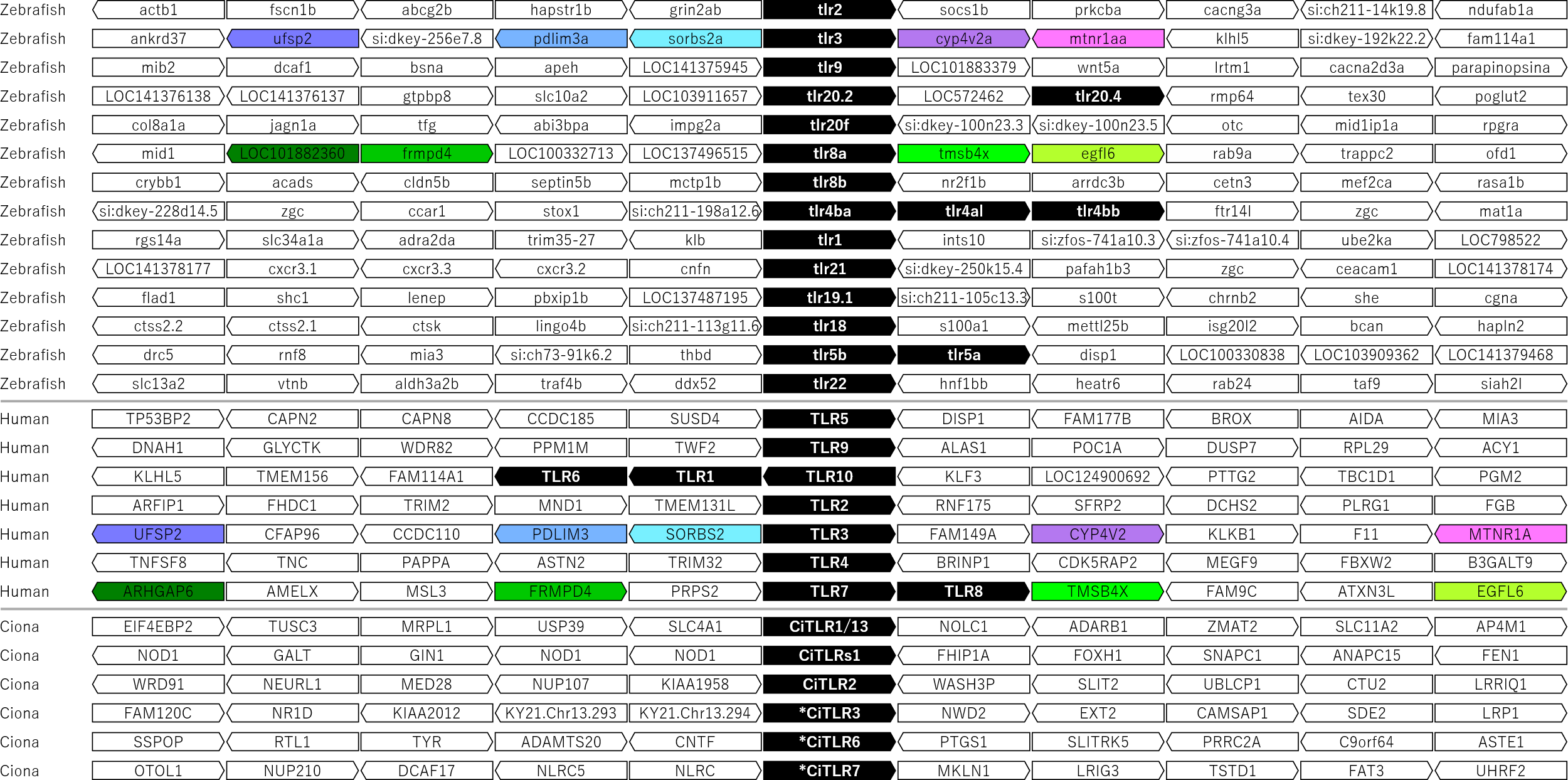
**

**Supplementary Figure 3 Comparative synteny analysis of *TLR* loci among representative chordates.** The five genes flanking the *TLR* locus on both sides are depicted in blocks with directional information. Genes for TLRs were depicted with black blocks. Genes with significant BLAST similarity (e-value ≤ 1e−20) are indicated by identical colors across species, representing regions of conserved synteny. Names for zebrafish and human genes were based on geneID in refseq. The names for *Ciona* genes were based on the blastp tophit against the refseq human proteome, which is released in Ghost Database.

**
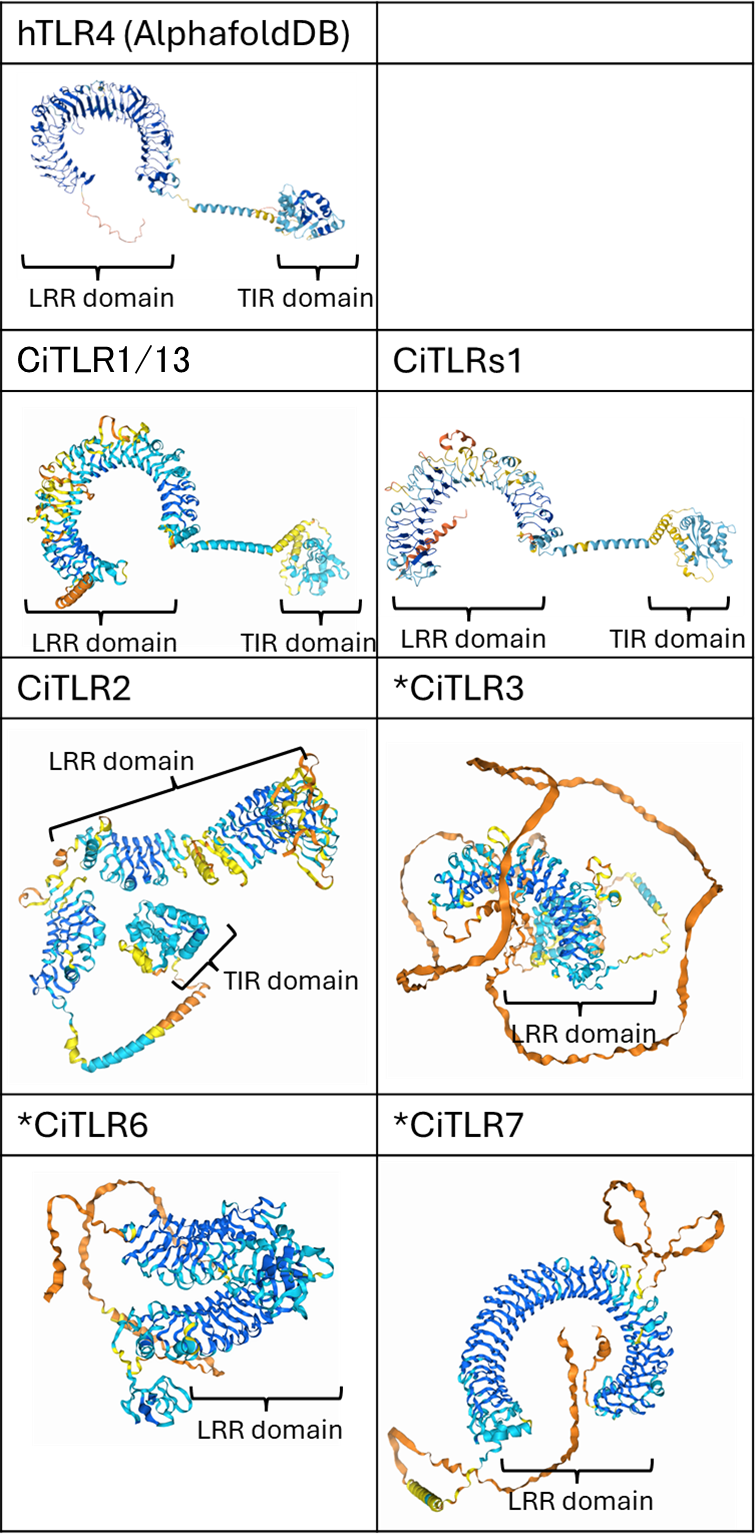
**

**Supplementary Figure 4.** AlphaFold3-generated putative three-dimensional structures of human TLR4 and CiTLRs.


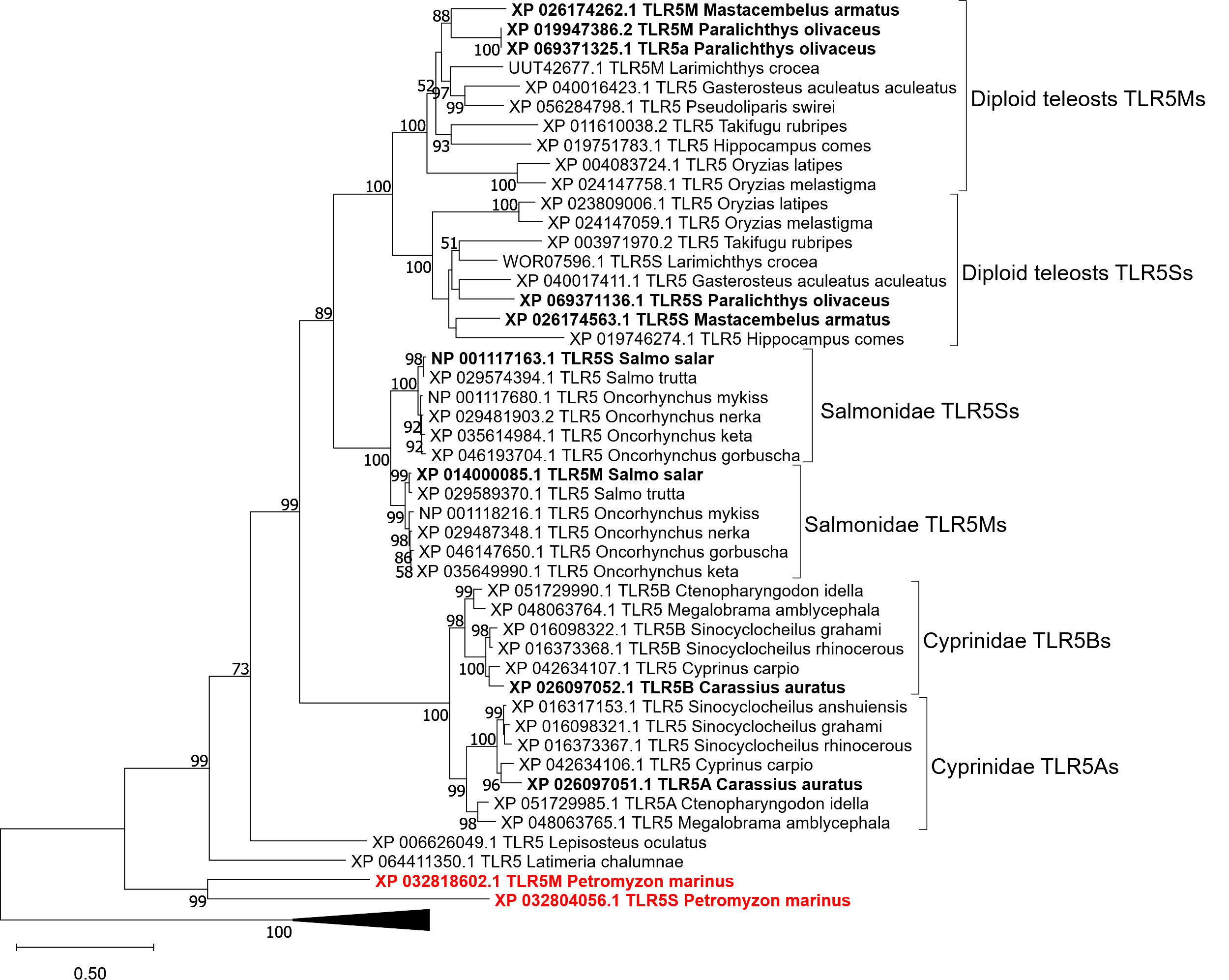


**Supplementary Figure 5. Molecular phylogenetic tree of the soluble and membrane-type TLR5s.** Lamprey’s PmTLR5S and PmTLR5M were shown in red and representative proteins in each clades were shown in bold.

## Materials and Methods for Supplementary information.

## Molecular Phylogenetic analysis

For the molecular phylogenetic analysis of Ciona TLRs, orthogroups (gene clusters sharing homology) were first identified using OrthoFinder (Emms and Kelly, 2019) (<https://www.ebi.ac.uk/interpro/download/InterProScan/>) with default parameters across ten vertebrates, three ascidians, one cephalochordate, one hemichordate and four echinoderms (Supplementary Table 2). From the resulting orthogroup sets, those containing CiTLRs were extracted for downstream analyses. To determine the orthogroup members possessing LRR domains, domain and motif searches were performed using InterProScan 6, including annotation by the SMART database. Protein sequences that were annotated as containing LRR domains were selected, and the amino acid sequences corresponding to these LRR regions were aligned using Clustal W-MPI 0.13. For the molecular phylogenetic analysis of PmTLR5S and PmTLR5M, teleosts TLR5s were collected by BLASTP using the query of PmTLR5S and PmTLR5M. The detected sequences with lower e-values than 10^-40^ were aligned using Clustal W-MPI 0.13. Then, maximum likelihood phylogenetic trees based on these alignmentswere constructed using FastTree 2.1.10 (Price, et al., 2010) (<https://morgannprice.github.io/fasttree/>) under a JTT matrix-based model with CAT approximation, and branch support values were estimated from 100 bootstrap replicates.

## Synteny analysis

For the synteny analysis, we first aimed to investigate the evolutionary conservation of genomic organization around TLR genes across vertebrates and tunicates. To this end, we extracted the genomic neighborhoods surrounding TLR genes, including five flanking genes on both sides, from the genome annotation files of *Danio rerio* (GCF_049306965.1), *Homo sapiens* (GCF_000001405.40), and *Ciona robusta* (HT version). Protein-coding sequences of the collected genes were used to construct BLAST databases for each species, and homologous sequences were identified across species using blastp. To visualize conserved synteny, gene pairs with an e-value less than or equal to 1 × 10⁻²⁰ were assigned the same color, thereby highlighting conserved genomic arrangements.

**References**

Emms, DM. and Kelly, S. OrthoFinder: phylogenetic orthology inference for comparative genomics. Genome Biology (2019) 20, 238. doi: 10.1186/s13059-019-1832-y.

Price, M.N.; Dehal, P.S.; Arkin, A.P. FastTree 2--approximately maximum-likelihood trees for large alignments. PLoS ONE (2010), *5*, e9490. doi: 10.1371/journal.pone.0009490
